# miR-148a suppresses human renal cell carcinoma malignancy by targeting AKT2

**DOI:** 10.3892/or.2022.8454

**Published:** 2022-11-28

**Authors:** Huiyan Cao, Zhiming Liu, Rong Wang, Xiaodong Zhang, Wenfa Yi, Guanyuan Nie, Yong Yu, Guolu Wang, Mingting Zhu

Oncol Rep 37: 147–154, 2017; DOI: 10.3892/or.2016.5257

Following the publication of the above paper, an interested reader drew to our attention that the western blot data shown in Fig. 7, the scratch-wound assay data in Fig. 2D and the cell invasion assay data in Fig. 2E were strikingly similar to data that had already been published in different articles by different authors from different research institutions, or which were already under consideration for publication elsewhere. Independently of the reader's enquiry, the authors contacted the Editorial Office to request that the paper be retracted on account of the fact that they were unable to reproduce the results presented in Fig. 2.

Owing to the fact that the contentious data in the above article were already under consideration for publication, or had already been published, elsewhere when it was submitted to *Oncology Reports*, and in line with the authors' own request, the Editor has decided that this paper should be retracted from the Journal. The Editor apologizes to the readership for any inconvenience caused.

